# Molecular Epidemiology and Complete Genome Characterization of H1N1pdm Virus from India

**DOI:** 10.1371/journal.pone.0056364

**Published:** 2013-02-15

**Authors:** Shashi Sharma, Gaurav Joshi, Paban K. Dash, Maria Thomas, Thimmasandra N. Athmaram, Jyoti S. Kumar, Anita Desai, Ravi Vasanthapuram, Ishan K. Patro, Putcha V. L. Rao, Manmohan Parida

**Affiliations:** 1 Division of Virology, Defence R&D Establishment (DRDE), Gwalior, India; 2 Department of Neurovirology, NIMHANS, Bangalore, India; 3 School of Studies in Neurosciences, Jiwaji University, Gwalior, India; Institut Pasteur, France

## Abstract

**Background:**

Influenza A virus is one of world’s major uncontrolled pathogen, causing seasonal epidemic as well as global pandemic. This was evidenced by recent emergence and continued prevalent 2009 swine origin pandemic H1N1 Influenza A virus, provoking first true pandemic in the past 40 years. In the course of its evolution, the virus acquired many mutations and multiple unidentified molecular determinants are likely responsible for the ability of the 2009 H1N1 virus to cause increased disease severity in humans. Availability of limited data on complete genome hampers the continuous monitoring of this type of events. Outbreaks with considerable morbidity and mortality have been reported from all parts of the country.

**Methods/Results:**

Considering a large number of clinical cases of infection complete genome based sequence characterization of Indian H1N1pdm virus and their phylogenetic analysis with respect to circulating global viruses was undertaken, to reveal the phylodynamic pattern of H1N1pdm virus in India from 2009–2011. The Clade VII was observed as a major circulating clade in phylogenetic analysis. Selection pressure analysis revealed 18 positively selected sites in major surface proteins of H1N1pdm virus.

**Conclusions:**

This study clearly revealed that clade VII has been identified as recent circulating clade in India as well globally. Few clade VII specific well identified markers undergone positive selection during virus evolution. Continuous monitoring of the H1N1pdm virus is warranted to track of the virus evolution and further transmission. This study will serve as a baseline data for future surveillance and also for development of suitable therapeutics.

## Introduction

Influenza A virus is known to cause an acute respiratory disease with a history of causing severe pandemics including the recent one by novel swine origin Influenza A virus (S-OIV). The property of virus subtype to mutate into variety of strains with differing pathogenic profile, eventually resulted in achieving higher fitness in a brief period. Influenza A virus is a member of family *Orthomyxoviridae.* Based on the antigenicity, virus may be classified into 16 Hemagglutinin (H1–H16) and 9 Neuraminidase (N1–N9) subtypes. Influenza A virus genome is composed of eight segments of single-stranded, negative-sense RNA and each of which encodes one or two proteins. The HA protein is critical for binding to cellular receptors and fusion of the viral and endosomal membranes. Replication and transcription of viral RNAs (vRNAs) are carried out by three polymerase subunits PB2, PB1, and PA, and the nucleoprotein (NP). Newly synthesized viral ribonucleoprotein complexes are exported from the nucleus to the cytoplasm by the nuclear export protein (NEP, formerly called NS2) and the matrix protein M1, and are assembled into virions at the plasma membrane. NA protein cleaves sialic acid residues on the host cell glycoproteins and glycolipids to which the HA proteins of newly assembled virions bind and, therefore plays an important role in the release of newly formed virions from the host cell membrane [1].

Several reports described both emergence and pandemic potential of the virus in the perspective of earlier pandemic influenza viruses of 1918 (H1N1), 1957 (H2N2) and 1968 (H3N2) through comparison of the available genetic sequence data [2]. The genetic analysis of the novel H1N1 virus isolated from a patient in California revealed that it was a recent reassortment of gene segments from both North American and Eurasian swine lineages. Since April 2009, the novel swine-origin influenza A (H1N1pdm) virus has rapidly spread across the globe. World Health Organization declared the outbreak a global pandemic in June 2009. The WHO global Influenza surveillance network has greatly contributed to the knowledge about circulating influenza viruses, including the emergence of novel strains [3]–[5]. This newly emerged virus represents a quadruple reassortment of two swine strains, one human strain, and one avian strain of influenza virus [6]. The largest proportion of genes comes from swine influenza virus strain (30.6% from North American swine influenza strains, 17.5% from Eurasian swine influenza strains), followed by North American avian influenza strains (34.4%) and human influenza strains (17.5%). Historically, pigs play an important role in interspecies transmission of influenza virus. Susceptible pig cells possess receptors for both avian (alpha 2–3-linked sialic acids) and human influenza strains (alpha 2–6-linked sialic acids). Presence of both receptors allow for the reassortment of influenza virus genes from different species, when a pig cell is infected with more than one strain [7]. The influenza A (H1N1pdm) has caused a considerable number of deaths within a short duration since its emergence [8].

The major symptoms of the disease is characterized by the sudden onset of high fever, chills, coughing, sore throat, muscle pain, severe headache, malaise, and inflammation of the upper respiratory tract and trachea, with general discomfort, but it rarely induces severe inflammatory lung diseases, including pneumonic involvement due to host innate and acquired immunity. Swine origin pandemic human influenza A virus (H1N1pdm) has spread rapidly around the world since its initial documentation in April 2009. According to last update (29 Jan 2010- update 85) of WHO in pandemic period H1N1pdm had spread to 209 countries and overseas territories, with 14711 deaths since the first reports of the virus in human in April 2009. In India the H1N1pdm virus is circulating through its emergence continuously and viral cases are being reported from different parts of the country in post pandemic phase [9]–[12]. Certain specific molecular markers predictive of adaptation to humans were found to be absent in the pandemic Influenza A 2009 (H1N1pdm) viruses suggesting that, previously unrecognized molecular determinants could be responsible for the transmission among humans. Several reports about the comparison of HA gene sequence with those of the earlier influenza pandemics have shown that human-specific markers supporting efficient transmissibility of these viruses in human are present in the H1N1pdm virus [1], [13]. Further, continuous monitoring of the evolution of this virus is advocated to track the mutations that may increase pathogenicity and/or transmissibility.

Understanding the virus evolution within India in relation to global diversification of the virus is also essential. So far, not much data is available on complete genome characterization of Indian H1N1pdm virus. The circumstances surrounding the emergence of this pathogen, and the factors that facilitated the initial cross-species transmission, are still not fully understood. It became apparent in the early days of the outbreak that the virus can be directly transmitted between humans. Among the various efforts made to evaluate, diagnose and implement the measures against the spread of virus, is the timely release of the genomic sequences from different viral isolates [14]. Keeping this in mind therefore, attempts were made to have adequate genome information to understand the true picture of novel H1N1pdm virus circulating in India. The present study was aimed to elucidate the complete genome sequence information of four recently circulating H1N1pdm virus isolated from different parts of India during 2010–2011. The phylodynamic pattern of H1N1pdm virus from 2009–2012 of global and Indian isolates was analyzed and the implication of resultant mutation due to selection pressure was also discussed in detail.

## Results

### Clinical Presentation of Suspected H1N1pdm Samples

**Figure 1:**
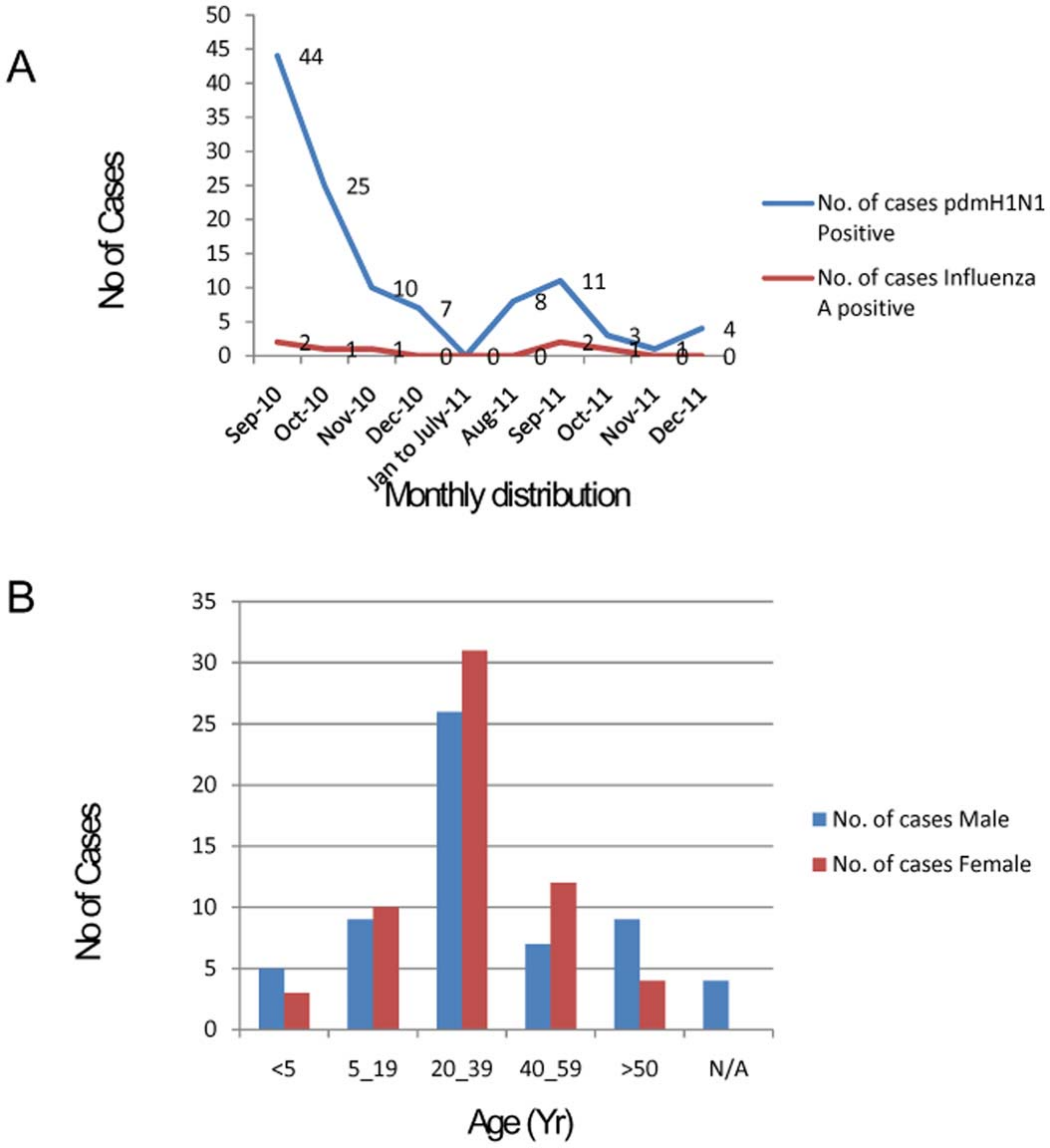
(A) Monthly trend of pandemic influenza A H1N1 and seasonal flu reported from September 2010 to December 2011. (B) Age and sex wise distribution of the influenza A H1N1 2010–2011 suspected ILI cases.

35 patients (WHO category C cases) were confirmed positive by CDC real time RT-PCR with positivity of 29.16%. The youngest case was a 6 months old female child. Monthly sample analysis profile revealed that 92.5% of the samples pertained to the period September-December 2010–2011, and the rest 7.5% of cases reported besides this period. 47.5% cases were seen amongst the age group of 20 to 39 years, while 15.83% cases were seen amongst the age group of 5–19 years. The median age of the samples investigated was 30 years (range 6 months- 76 years). 6.66% of the patients were under age 5 and 10.83% were more than 54 years old. The female/male ratio for H1N1pdm in different age groups were significantly greater than 1. No patient was previously vaccinated, however oseltamivir was started after 5 days in 30% of the cases. An overall case fatality rate was 8.33% with 10 deaths. Maximum deaths were seen in younger age group (7–25 years) with increased case fatality rate of 15% in 2011. Death in complicated cases occurred between 24–48 hours of report to hospitals. The clinical history revealed that all the patients had suffered from fever (>38.0°C). Other prominent clinical symptoms include fever (axilla, Oral) (80%), cough (42%), sore throat (38%), nasal catarrh (75%) and shortness of breath (66%). Monthly and age wise distribution of suspected patients is summarized in Figure 1.

### Laboratory Diagnosis of H1N1pdm Samples

Out of 120 suspected samples, 35 (29.16%) were positive for pandemic Influenza A H1N1 and 7 (5.83%) were positive for Influenza A (Seasonal virus). The cases of H1N1pdm started rising from September 2010 with maximum number of cases (n = 44). All the samples were diagnosed by WHO approved CDC Real time RT-PCR using 4 sets of primer and probes. Samples found positive for all the four probes viz. Influenza A, Swine Influenza A, Swine H1, RNase P (Inf A, swA, swH1, RNP) were declared positive for H1N1pdm virus. Each lot of samples were tested with a positive confirmed H1N1pdm cell culture RNA as positive control and healthy throat swab sample RNA as negative control. Detailed features including clinical presentations of H1N1pdm positive samples were summarized in Table 1.

**Table 1:** Details of positive cases for H1N1pdm virus during investigation of suspected samples from 2010–2011.

Sl.No	Details of clinical samples	Sampling Date	Age(Yr/month)	Gender	Ct value(swH1)	Clinical Symptoms
1	DRDE 01_11	24/08/11	20 Yr	F	34	Fever, cough, shortness in breath
2	DRDE 02_11	24/08/11	22 Yr	M	38	Cough
3	DRDE 09_11	07/09/11	25 Yr	M	39	Cough, difficulty in Breathing
4	DRDE 10_11	08/09/11	16 Yr	F	40	Fever, difficulty in breathing
5	DRDE 11_11	12/09/11	43 Yr	F	39	Fever, cough, shortness in breath
6	DRDE 19_11	01/12/11	23 Yr	M	37	Fever, cough, sore throat, shortness in breath
7	DRDE-01_10	02/09/2010	28 yr	M	30	Fever, cough, shortness in breath
8	DRDE-02_10	05/09/2010	54 yr	M	38	Fever, cough, sore throat
9	DRDE-03_10	05/09/2010	55 yr	F	36	Fever, shortness in breath
10	DRDE-05_10	08/09/2010	46 yr	M	35	Fever (Oral), nasal catarrh
11	DRDE-07_10	09/09/2010	7 yr	M	23	Fever, nasal catarrh, shortness in breath
12	DRDE-08_10	10/09/2010	25 yr	M	33	Fever (Oral), cough, difficulty in breathing
13	DRDE-16_10	15/09/2010	35 yr	M	32	Fever, cough, sore throat,shortness in breath
14	DRDE-17_10	15/09/2010	37 yr	M	32	Fever (Oral), cough, difficulty in breathing
15	DRDE-18_10	15/09/2010	11 yr	M	32	Fever (Oral), nasal catarrh
16	DRDE-21_10	15/09/2010	25 yr	M	32	Fever, nasal catarrh, shortness in breath
17	DRDE-22_10	16/09/2010	26 yr	M	30	Fever, cough, sore throat shortness in breath
18	DRDE-25_10	17/09/2010	25 yr	F	30	Fever, cough
19	DRDE-26_10	17/09/2010	30 yr	F	38	Fever, cough
20	DRDE-31_10	21/09/2010	28 yr	F	29	Fever, nasal catarrh, shortness in breath
21	DRDE-37_10	21/09/2010	30 yr	M	35	Fever
22	DRDE-39_10	24/09/2010	72 yr	M	32	Nasal catarrh, shortness in breath
23	DRDE-46_10	07/10/2010	45 yr	F	36	cough, sore throat
24	DRDE-47_10	12/10/2010	7 yr	F	25	Fever (Oral), nasal catarrh
25	DRDE-48_10	14/10/2010	49 yr	F	36	cough, difficulty in breathing
26	DRDE-49_10	14/10/2010	30 yr	M	34	Fever, nasal catarrh, shortness in breath
27	DRDE-50_10	15/10/10	40 yr	F	36	Fever, cough, shortness in breath
28	DRDE-51_10	15/10/2010	6 mth	F	34	Fever, cough, sore throat,shortness in breath
29	DRDE-52_10	15/10/2010	3 yr	M	35	Fever (Oral), cough, difficulty in breathing
30	DRDE-53_10	15/10/2010	3 yr	M	34	Fever, cough, sore throat,shortness in breath
31	DRDE-55_10	16/10/2010	23 yr	M	36	Fever (Oral), cough, difficulty in breathing
32	DRDE-56_10	17/10/2010	28 yr	M	36	Fever, cough, shortness in breath
33	DRDE-57_10	19/10/2010	45 yr	F	36	Fever, cough, sore throat,shortness in breath
34	DRDE-62_10	26/10/2010	28 yr	M	33	Fever (Oral), cough, difficulty in breathing
35	DRDE-63_10	2/11/2010	76 yr	F	35	Fever, cough, sore throat,shortness in breath

### Isolation and Identification of H1N1pdm Virus

**Figure 2:**
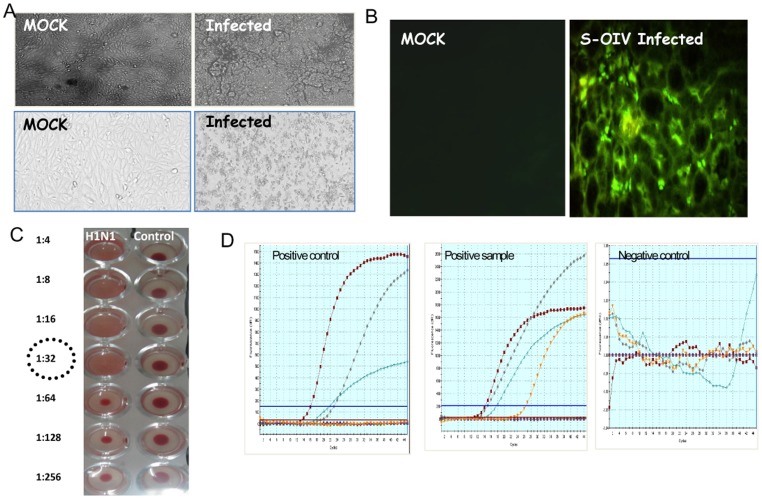
Confirmation of H1N1pdm virus. (**A**) Microscopic photograph of healthy and Influenza A (H1N1pdm) virus infected Madin Darby Canine Kidney Cells. (**B**) Immunofluorescence assay. (**C**) Haemagglutination assay. (**D**) WHO CDC Real-Time PCR amplification. Real time amplification curve of positive clinical samples showing amplification of all four probes.

Three selected positive samples were attempted for the H1N1pdm virus isolation in MDCK cells through three blind passages. Initially, H1N1pdm virus infection in MDCK cells was analysed microscopically for the appearance of prominent cyotopathic effects (granulation, clustering and finally total detachment from the adherent surface) till 48–72 hpi (Figure 2A). Infected cell culture supernatant was harvested at this stage and used for further identification and complete genome characterization. Hemagglutination (HA) titre with guinea pig RBC was determined in infected culture supernatant i.e. the highest dilution at which hemagglutination occurred. The HA titre was found 16–32 for the four different isolates used in this study (Figure 2C). Immunofluorescence test was performed to observe localization of the intracellular H1N1pdm virus using anti-pdmH1N1 HA polyclonal antibody (GenScript, USA). Bright apple green fluorescence was observed in H1N1pdm virus infected cells whereas no fluorescence was observed in mock infected MDCK cells (Figure 2B). Virus isolation was also confirmed at genomic level at different passage level with WHO approved CDC Real time RT-PCR (Figure 2D).

### RT-PCR for the Amplification of Complete Genome

A total of 4 representative H1N1pdm viruses comprising two 2010 isolates (one each from Gwalior and Bangalore) and two 2011 isolates (Fatal cases) of Gwalior were selected for complete genome amplification. These samples at passage level three in MDCK cells were used for the purpose and were subjected to complete genome amplification. The editing and alignment of sequences of overlapping fragments led to sequence information of complete genome. Complete genome (concatenated eight gene segments) of isolates deciphered was 13158nt. The sequences (PB2, PB1, PA, HA, NP, NA, MP and NS) for all the four isolates were deposited in GenBank with following accession numbers JF265678, JF265677, JF764085, JF764086, JF265676, JF265675, JF293316, JF293315, JF265674, JF265673, JF265672, JF265671, JF764082, JF510037, JF764083, JF764084, JQ 319657-58, JX 262203-04, JX 262207-08, JX262205-06, JX 262209-10, JX 262201-02, JX 262211-12, JX 262213-14.

### Analysis of the Concatenated Complete Genome of the Indian H1N1pdm Virus

The genome sequences of representative Influenza A (H1N1pdm) viruses of diverse geographical origins were retrieved from NCBI GenBank database from the period of 2009–2012 (Table 2). Comparative sequence analysis with A/California/04/2009 H1N1pdm prototype strain of the four Indian isolates revealed >98% (ranged between 98.9–99.8%) nucleotide identity in the different gene segments. The percent amino acid divergence (PAD) within each gene segment of four Indian isolates ranged from 0% (in M2) −1.2% (in NP). The PAD within HA genes of four Indian isolates sequenced in this study were found in a range of 0.4–0.9%. All the four viruses sequenced in this study revealed >99% amino acid sequence identity for the HA protein of previous Indian H1N1pdm virus isolated in 2009 (Pune/NIV6447/2009, Pune/NIV8489/2009, Blore/NIV236/2009, Blore/NIV310/2009, Mum/NIV5442/2009).

**Table 2:** Details of the genome sequences of the H1N1pdm virus isolates retrieved and investigated in the whole genome and complete HA gene based phylogenetic analysis in this study.

Sl.No	Strain	Year	Geographic origin	clade
1	California/04	2009	USA	I
2	California/07	2009	USA	I
3	California/06	2009	US	II
4	CanadaAB/RV1644	2009	North America	II
5	Colarado/03	2009	USA	II
6	Nebraska/02	2009	USA	II
7	Brawley/40081	2009	US	II
8	Carven/WR0019	2009	US	II
9	Indiana/09	2009	US	II
10	Minnesota/02	2009	US	II
11	Hamburg/4	2009	Europe	II
12	Kansas/03	2009	US	II
13	Netherlands/602	2009	Europe	II
14	Nanjing/2	2009	China	III
15	England/195	2009	UK	III
16	Wisconsin/629D00022	2009	US	III
17	Sichuan/1	2009	China	III
18	Vladivostok/01	2009	Russia	III
19	SantoDomingo/0573N	2009	America	III
20	South Carolina/09	2009	America	III
21	New York/3177	2009	USA	III
22	Moscow WRAIR4316N	2011	Russia	III
23	Amagasaki/1	2009	Japan	IV
24	Sakai/1	2009	Japan	IV
25	Kobe/1	2009	Japan	IV
26	Osaka/1	2009	Japan	IV
27	Himeji/1	2009	Japan	IV
28	Korea/01	2009	Korea	IV
29	Beijing/3	2009	China	V
30	Beijing/501	2009	China	V
31	Hunan/SWL3	2009	China	V
32	Utsunomiya/1	2009	Japan	V
33	CanadaPQ/RV1758	2009	Canada	V
34	NewYork/4735	2009	USA	V
35	Wisconsin/629D00008	2009	USA	V
36	India-Pune/NIV6196	2009	India	VI
37	NewYork/3324	2009	USA	VI
38	Shanghai/143T	2009	China	VI
39	SilverSpring/SP509	2009	US	VI
40	Taiwan/T1773	2009	Taiwan	VI
41	Mexico In DRE3740	2011	US	VII
42	Thailand CU-H2911	2011	Thailand	VII
40	Sydney DD3–58	2011	Australia	VII
43	Missouri NHR C0001	2011	USA	VII
44	Tomsk IIV-19	2012	Russia	VII
45	Cheboksary IIV-92	2011	Russia	VII
46	Brazil AVS08	2011	Brazil	VII
47	California NHRC0001	2011	USA	VII
48	Boston DOA14	2011	USA	VII
49	Taiwan 1018	2011	Taiwan	VII
50	San Salvador 0196T	2009	USA	VII
51	Shiga/3	2009	Japan	VII
52	Shizuoka/759	2009	Japan	VII
53	CherryPoint/WR0100	2009	USA	VII
54	NewBern/WR0670	2009	US	VII
55	MexicoCity/WR1100N	2009	Mexico	VII
56	Mexico In DRE3740	2011	Mexico	VII
57	Nanjing/3	2009	China	VII
58	Shanghai/1	2009	China	VII
59	Ohio/07	2009	USA	VII
60	Denmark/523	2009	Europe	VII
61	pune/NIV8489	2009	India	VII
62	pune/NIV6447	2009	India	VII
63	Blore/NIV310	2009	India	VII
64	Mum/NIV5442	2009	India	VII
65	Delhi/NIV3610	2009	India	VII
66	Blore/NIV236	2009	India	VII
67	Hyd/NIV51	2009	India	VII
68	Mum/NIV9945	2009	India	VII
69	pune/NIV10278	2009	India	VII
70	pune/NIV9355	2009	India	VII
71	Delhi/NIV3704	2009	India	VII
72	Omsk/02	2009	Russia	VII
73	Netherland/2631	2010	Europe	VII
74	Cambodia/U127	2010	Asia	VII
75	Assam/2220	2009	India	VII
76	Assam/2590	2010	India	VII
77	Finland/65	2011	US	VII
78	Volgograde/CRIE-DMV	2011	Russia	VII
79	Bangkok/INS520	2010	Thailand	VII
80	Shanghai/3162T	2011	China	VII
81	St. Petersburg/CRIE-GOVM	2011	Russia	VII
82	Rio Grande do sul/361	2011	Brazil	VII
83	Nizhnii Novgorod/CRIE-BLM	2011	Russia	VII
84	District of Columbia/WRAIR313	2011	US	VII
85	**Bangalore-NIM**	**2010**	**India**	**VII**
86	**GWL-DSC**	**2010**	**India**	**VII**
87	**GWL-01**	**2011**	**India**	**VII**
88	**GWL-02**	**2011**	**India**	**VII**

Note: The isolates in bold font are sequenced in this study.

### Phylogenetic Analysis

**Figure 3:**
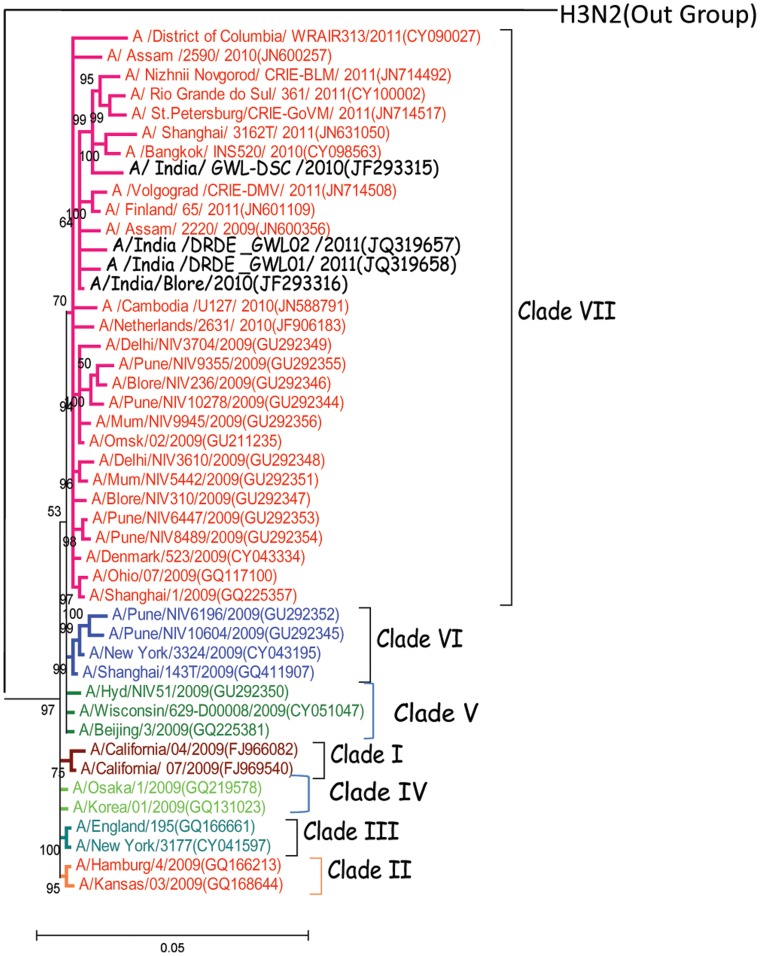
Phylogenetic tree among H1N1pdm viruses generated by Bayesian method based on Full HA gene (1701 nucleotides). Each strain is highlighted with virus subtype, country of origin, strain name, year of isolation and accession number in parenthesis. Each clade is defined by long branch and nodes supported by high Bayesian posterior probability (BPP) values (90%). Scale bar indicates number of nucleotide substitutions per site.

**Figure 4:**
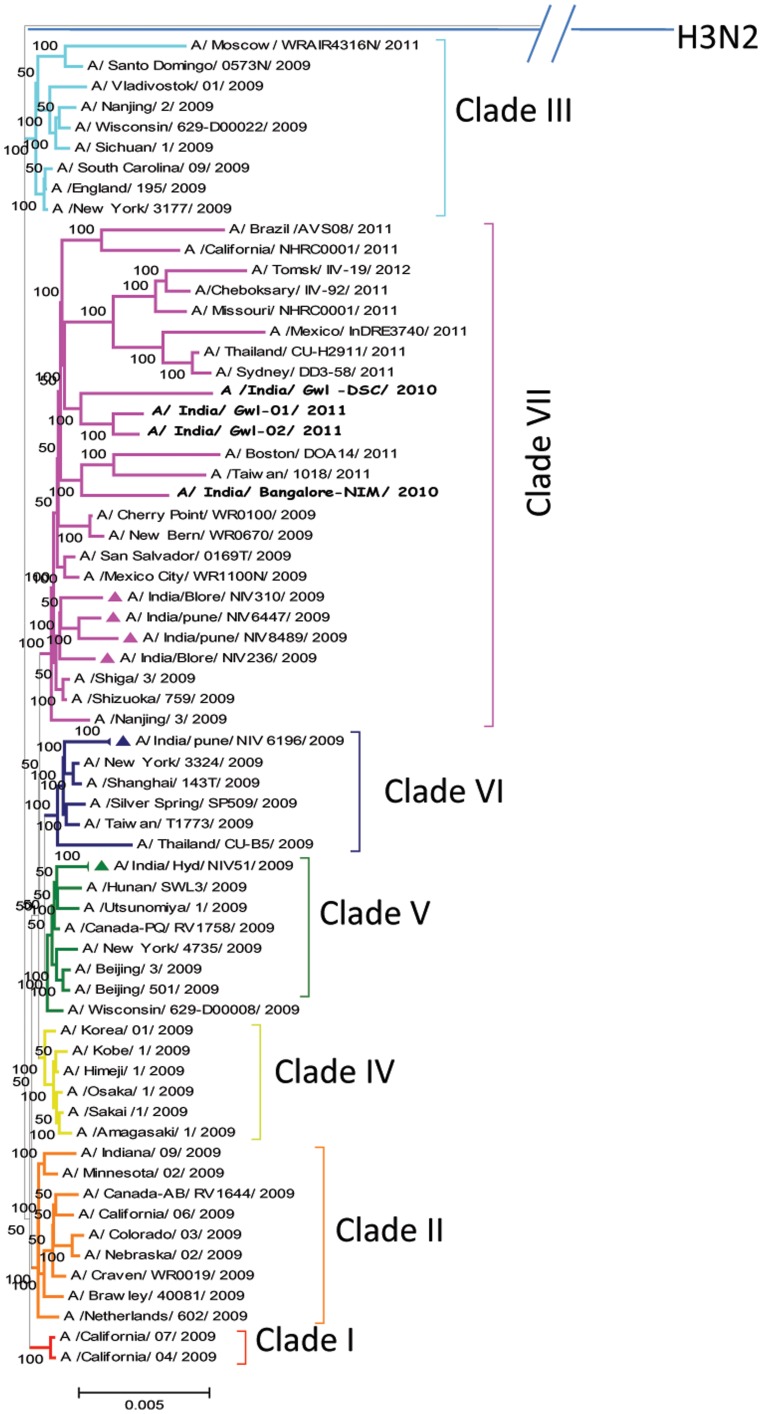
Phylogenetic tree of concatenated whole genome of representative global H1N1pdm viruses including four Indian viruses sequenced in this study generated by Bayesian method. Each strain is abbreviated with virus subtype, country of origin, strain name and year of isolation in parenthesis. Scale bar indicates number of nucleotide substitutions per site. The Indian isolates sequenced in this study are highlighted in different font in clade VII. Other Indian isolates are highlighted by solid diamond in respective clades. Each clade is defined by long branch and nodes supported by high Bayesian posterior probability (BPP) values (100%).

Extensive phylogenetic analysis based on concatenated whole genome sequences (13158 nt; n = 65) and full HA gene (1701 nt; n = 45) of representative H1N1pdm viruses sampled between 2009–2012 from different geographical regions along with the Indian isolates revealed seven distinct clades (Figure 3 and Figure 4 ). Both the phylogenetic analysis revealed the same topology. All the four Indian isolates sequenced in this study formed a close branch and grouped into clade VII. This clade VII was represented by maximum number of isolates from geographically diverse areas. The prototype A/California/04/2009 and A/California/07/2009 from California belong to clade I. Clade II is represented by H1N1pdm virus isolated from California, Canada, Netherlands, and United States. Clade III is represented by H1N1pdm virus isolated from England, Russia, China, and the United States. Clade IV is represented by H1N1pdm virus isolated from two East Asian countries, Korea and Japan. Clade V is represented by H1N1pdm virus isolated from Canada, China, Japan, the United States (mainly Wisconsin isolates) along with India. Clade VI is represented by H1N1pdm virus isolated from China, Japan with new additions from Taiwan, Thailand, India and United States. The clade VII, which is the largest clade is represented by H1N1pdm virus isolated from Japan, Mexico, China, Asia and several states of the USA. H3N2 virus was taken as an outgroup for rooting the tree during phylogenetic analysis. Almost all the representative circulating H1N1pdm viruses from India were included in the phylogenetic analysis from 2009–2012.

### Analysis of Individual Gene Segments

Comparison of individual gene segment at protein level with respect to A/California/04/2009 (H1N1pdm prototype strain) and A/India/Pune/NIV6447/2009 (previously sequenced Indian strain) revealed a total of 73 substitutions scattered throughout the eight gene segments in four Indian viruses sequenced in this study. The sequence alignment revealed amino acid replacement throughout the aligned region. The 47 major/important non-conservative and clade specific amino acid substitutions among H1N1pdm virus (sequenced in this study) vis-a-vis prototype California/04/2009 and A/India/pune/NIV6447/2009 are shown in Table 3. The clade specific mutations in different genes, NP: V100I; NA: V106I; HA: P100S, T214A, S220T, I338V; NS1:I123V; PA: P224S were reported amongst the four Indian isolates. The M2 protein of four Indian isolates did not have any mutation compared to prototype California/04/2009 strain. P100S substitution observed in all Indian isolates was located in the antigenic site E and S202T substitution observed in one Indian isolate (A/India/GWL_DSC/2010) was located in antigenic site B. Further, substitution S220T (in all four Indian viruses); N245I (in one Indian virus A/India/GWL/01/2011) was found in the vicinity of site D [22]. The residue position for the HA is the numbering considered inclusive of the signal peptide. All the Indian viruses possessed residue H275 a known marker for sensitivity to the neuraminidase inhibitor, Oseltamivir. The four Indian H1N1pdm viruses had the genetic marker 31N in the M2 gene suggesting Amantadine resistance.

**Table 3:** Description of major/important non-conservative and clade specific amino acid substitutions among the four Indian H1N1pdm virus (sequenced in this study) compared to prototype H1N1pdm strain (California/04/2009) and other Indian (A/Pune/NIV6447/2009) virus strain (sequenced previously).

Gene segment	Residue number *	A/California/04/2009	A/Pune/NIV6447/2009	A/India/Bangalore_NIM/2010	A/India/GWL_DSC/2010		A/India/Gwl_01/2011	A/India/Gwl_02/2011
PB2	216	*R*	*R*	*R*		*R*	*G*	*R*
	241	*E*	*E*	*E*		*G*	*E*	*E*
	288	*Q*	*Q*	*Q*		*P*	*Q*	*Q*
	309	*D*	*D*	*N*		*D*	*D*	*D*
	340	*K*	*K*	*K*		*T*	*T*	*T*
	439	*Q*	*Q*	*H*		*H*	*Q*	*Q*
	441	**D**	**D**	**H**		**H**	**D**	**D**
	588	**T**	**T**	**I**		**T**	**I**	**I**
PB1	39	**T**	**T**	**I**		**T**	**T**	**T**
	151	*R*	*R*	*G*		*R*	*R*	*R*
	152	**S**	**S**	**L**		**S**	**S**	**S**
	229	*K*	*K*	*K*		*I*	*K*	*K*
	299	**S**	**S**	**F**		**S**	**S**	**S**
	307	*T*	*T*	*P*		*T*	*T*	*T*
	323	*T*	*T*	*T*		*K*	*T*	*T*
	435	**I**	**I**	**T**		**I**	**T**	**T**
PA	30	**I**	**I**	**I**		**I**	**N**	**N**
	142	*K*	*K*	*K*		*K*	*N*	*N*
	168	*R*	*R*	*R*		*T*	*R*	*R*
	224	*P*	*S*	*S*		*S*	*S*	*S*
	514	*D*	*D*	*D*		*Y*	*D*	*D*
	644	*S*	*S*	*C*		*S*	*S*	*S*
HA	4	**I**	**T**	**I**		**I**	**I**	**I**
	100	*P*	*S*	*S*		*S*	*S*	*S*
	202	S	S	S		T	S	S
	214	**T**	**A**	**A**		**A**	**A**	**A**
	220	S	T	T		T	T	T
	245	**N**	**N**	**N**		**N**	**I**	**N**
	246	**Y**	**Y**	**Y**		**Y**	**N**	**Y**
	338	I	V	V		V	V	V
	391	**E**	**E**	**K**		**K**	**K**	**K**
	435	**I**	**I**	**I**		**T**	**I**	**I**
	442	*L*	*L*	*L*		*L*	*R*	*L*
NP	100	V	I	I		I	I	I
	194	**I**	**I**	**T**		**I**	**I**	**I**
	197	*I*	*I*	*H*		*I*	*I*	*I*
	232	*T*	*P*	*T*		*T*	*T*	*T*
NA	30	I	V	I		I	I	I
	106	V	I	I		I	I	I
	248	*N*	*D*	*N*		*N*	*D*	*D*
NS1	55	*E*	*E*	*Q*		*E*	*E*	*E*
	71	**E**	**E**	**E**		**K**	**E**	**E**
	123	I	V	V		V	V	V
	200	*R*	*R*	*G*		*R*	*R*	*R*
	211	*R*	*R*	*G*		*R*	*R*	*R*
NS2	43	*D*	*D*	*G*		*D*	*D*	*D*
	52	**M**	**M**	**M**		**M**	**T**	**T**

Note: Major non conservative changes involving basic to acidic amino acid are written in bold font and also underlined; The hydrophobic to hydrophilic amino acid substitutions and vice-versa are written in bold font. The substitutions involving charged residues to uncharged residues; cyclic to acyclic and vice versa are written in italics. The clade specific substitutions (NP:V100I; NA:V106I; NS1:I123V; HA:S220T, I338V) are written in normal font.

* The residue position for the HA is the numbering considered inclusive of signal peptide.

### Selection Pressure Analysis

Selection pressure analysis of HA, NA and MP gene of 72 global H1N1pdm virus strains revealed 18 positively selected sites. Integrated analysis was performed for differential selection pressure acting on HA (566 codons), NA (469 codons), M1 (252 codons) and M2 (97 codons) proteins. Positive selection on HA gene was stronger than NA, M1 and M2 protein gene. In total 11 HA, 3 NA, 2 M1 and 2 M2 sites were found under positive selection by at least two methods (Table 4). Out of 11 HA sites, 2 positions were located in signal peptide, 4 sites in HA1 and 5 sites in HA2. Position 151, 222 and 239 were situated within a known B-cell antigenic region. 3 sites (30, 248 and 386) in NA gene were found to be positively selected. Analysis of matrix protein gene revealed 2 sites each in M1 (28, 181) and M2 (10, 26) to be under positive selection. A specific selection pressure analysis for Indian isolates (n = 17) for HA and NA gene revealed 3 sites in HA and 2 sites in NA gene under positive selection (Table S3). Out of these S220T (HA) and N248D (NA) were earlier attributed to clade VII specific substitutions [19], [21].

**Table 4:** Selection pressure analysis of HA protein (566 codons); NA protein (469 codons), M1 Protein (252 codons) and M2 Protein (97 codons) of H1N1pdm virus using SLAC, FEL,REL,MEME and FUBAR methods. (www.datamonkey.org).

Protein	Codon	SLAC		FEL		REL		MEME		FUBAR	
		dN-dS	p-value	dN-dS	p-value	dN-dS	Bayes Factor	ω^+^	p-value	dN-dS	Post. Pr.
**HA**	8	2.652	0.2	36.57	0.059	0.121	247.43	>100	0.078	1.081	0.828
	13	1.782	0.4	21.03	0.26	0.115	181.65	>100	0.155	0.297	0.694
	49	6.666	0.667	80.78	0.27	0.018	13.525	>100	0.134	0.095	0.555
	**151**	**1.781**	**0.444**	**20.97**	**0.27**	**0.115**	**181.19**	**>100**	**0.282**	**0.298**	**0.694**
	**222**	**1.779**	**0.48**	**19.85**	**0.326**	**0.114**	**174.110**	**>100**	**0.326**	**0.217**	**0.672**
	239	3.960	0.242	54.494	0.111	0.138	358.00	>100	0.137	2.89	0.965
	391	2.574	0.365	34.89	0.108	0.136	2702.7	>100	0.134	1.27	0.880
	436	2.648	0.991	40.43	0.768	0.121	257.96	>100	0.035	0.304	0.699
	442	1.405	0.423	19.61	0.165	-0.09	14.71	>100	0.198	0.243	0.642
	477	1.696	0.49	22.94	0.25	0.116	192.76	>100	0.269	0.34	0.69
	537	1.79	0.441	22.211	0.206	0.115	188.67	>100	0.228	0.349	0.710
**NA**	30	28.3	0.22	154.64	0.052	4.69	47704.3	>100	0.044	5.528	0.991
	248	20.343	0.369	88.97	0.187	4.385	1604.05	>100	0.42	2.66	0.932
	386	13.58	0.514	65.74	0.221	1.738	84.78	>100	0.241	0.95	0.80
**M1**	28	15.738	0.426	72.66	0.232	-0.688	1.0	>100	0.250	0.545	0.702
	181	15.6	0.41	70.76	0.23	-0.671	1.0	>100	0.265	0.54	0.718
**M2**	10	10.285	0.450	236.62	0.235	0.949	67.71	>100	0.252	1.795	0.890
	26	5.167	0.668	143.73	0.268	0.373	7.499	>100	0.281	0.848	0.761

Note: The sites found under positive selection by atleast two methods are shown*. Site present in B-cell epitope region are highlighted in bold font.

* **Significance value.**

SLAC P value–0.5.

FEL P value- 0.25.

REL Bayes factor- 50.

MEME P value- 0.1.

FUBAR Posterior probability- 0.9.

## Discussion

Transmission of pandemic Influenza virus is persisting in many continents but current activity levels are low in Asia. Recent peaks in the activity were noted during early 2010 in northern India, Nepal and Sri Lanka. Influenza activity remained stable but elevated in western India, continued to decline substantially in Northern India, and remained low overall in Southern and Eastern India [15]. This virus was generated by multiple reassortment events, and each of its precursor gene segments has circulated in swine for more than 10 years. Infection of swine with H1N1/2009 virus has been observed in multiple countries. But, because of a paucity of systematic surveillance of swine influenza worldwide the question remains whether H1N1/2009 will become established in swine and become a reservoir of reassortment that may produce novel viruses of potential threat to public health [16]. The H1N1/2009 virus has remained antigenically and genetically stable and are relatively low virulence in humans since its detection in April 2009. Most genetic changes in H1N1pdm to date have not been clearly linked to changes in antigenicity, disease severity, antiviral drug resistance, or transmission efficiency. However, rapid evolution rate characteristic of influenza viruses suggest that changes in antigenicity are inevitable in future [17]. With the number of reported pandemic cases of H1N1 virus in many parts of the world and continued viral persistence in India and nearby countries (Nepal, Sri Lanka, Bangladesh), elevated activity has given an urgent need to track the global dispersion of this virus in humans.

In this particular study, the main focus was complete genome characterization of the circulating isolates of northern India (Gwalior region) and to decipher conservative and non conservative substitutions, its comparative analysis with respect to other Indian and global circulating H1N1pdm isolates. The continued circulation of virus in particular region from 2009-till date is also a serious concern and required in depth investigation. With the determined objective of molecular investigation of circulating H1N1pdm virus, Influenza like illness (ILI) in suspected clinical samples from Gwalior, India during 2010–2011 were investigated. The clinical picture of the patients revealed the same pattern as was reported in 2009 [18] but there was an increase in number of H1N1pdm cases in 2010. It was revealed during the study that the virus has affected all the age groups with the highest in young age group. The numbers of females were affected more than males during the period under observation. Fatality ratio (5.83%) was found prominently high in young persons. Young groups have least experience of influenza A (H1N1pdm) virus and are recognized as potential source in the transmission of influenza. It is also possible that propensity to consult doctor is greatest in younger age groups. However, in 2011 the numbers of positive cases were higher in young age group of 18–28 Yr. The possible reason of higher cases in 2011 may be increase in viral virulence and its better adaptation in the region, which may become severe in the coming years.

In this study four Indian isolates that are confirmed by virus specific CPE, HA, IFT as well as CDC Real time RT-PCR were selected for complete genome characterization. The nucleotide sequence analysis revealed that there is no significant difference among viruses recovered from two different places and of different years from India. Diversity of the Indian isolates at the amino acid level with respect to the prototype strain and within the Indian isolates was found to be maximum in the HA and NP gene. Substitution S220T (HA) specific to clade VII isolates was adequate to lineate the isolates in HA-based phylogeny. Most of the amino acid changes were conservative, involving interchanges of amino acids having same physicochemical properties. However, few major non-conservative changes between Indian isolates were also observed. Compared to the prototype strain, glutamic acid was replaced by a strongly basic amino acid lysine at position 391 (HA) among the four Indian H1N1pdm virus and at the position 71 (NS1) in one Indian H1N1pdm virus sequenced in this study. Two important non conservative substitutions involving acidic aspartic acid to basic histidine at position 441(PB2) in two Indian H1N1pdm virus and cyclic proline to acyclic serine at position 100(HA) among the four Indian H1N1pdm virus were also recorded. Similar non conservative substitutions involving shift in amino acids were also recorded in other gene segments. However, the significance of these substitutions need to be addressed.

To identify genetic lineage of H1N1pdm virus, phylogenetic analysis was conducted for concatenated whole genome sequences retrieved from GenBank from 2009–2012 including all the available H1N1pdm whole genome from India sequenced till date. Whole genome and full HA based phylogenetic analysis revealed existing seven discrete clades of H1N1pdm virus circulating globally. Both the trees based on genome information comprised of all representative H1N1pdm clades from diverse geographical origin which included maximum number of representative H1N1pdm from all the affected areas. Both the trees yielded similar topologies, with characteristic distribution of H1N1pdm isolates into seven distinct clades. Maximum numbers of isolates were grouped into clade VII. The clade I included prototype California/04 and California/07 virus isolated first during H1N1pdm [19]. All Indian isolates (2009–2011) were grouped in clade VII except Hyd/NIV51/2009 and Pune/NIV6196/2009, Pune/NIV10604/2009 (HA gene phylogeny) virus isolated during initial pandemic phase grouped into clade V and VI respectively [20]. Clade VII is identified as predominant circulating clade in India, Asia as well as globally [19]. Phylogenetic analysis of all Indian H1N1pdm complete genome sequenced so far demonstrated that earliest isolate from Hyderabad (A/India/Hyd/NIV51/2009) during initial pandemic phase was a clade V isolate. Two other isolates from Pune during later pandemic phase (A/India/pune/NIV6196/2009, A/India/pune/NIV10604/2009) belonged to clade VI. Both the cases were not directly associated with any foreign travel history that is why it is not clear whether the clade evolved within the country or were imparted into the country. All other Indian isolates from last pandemic phase to post pandemic phase belonged to clade VII. Two initial Indian isolates belonging to clade VII had a foreign travel history and thus may be indicative of the fact that clade VII was introduced from an external source [21]. Therefore it may be possible clade VII is favourably selected as dominant H1N1pdm lineage in India.

Influenza viruses comprise of segmented viral genome, and are more prone to genetic reassortment during mixed infections. Hence the circulating H1N1pdm strains also evolve and may favourably be selected with higher fitness at a particular time point. It is most likely that the H1N1pdm strains were also undergone similar evolutionary process and the viruses of higher fitness were favourably selected over time. The selection pressure analysis revealed 18 positively selected sites in major surface proteins of Influenza A (H1N1pdm) virus i.e. HA, NA and matrix proteins. Since these proteins plays crucial role in the attachment, assembly, release of the virus, these substitutions might have played important role in making these isolates more transmissible. Differential selection analysis also supported the pandemic 2009 strains being subject to distinctive selection compared to their progenitors [21]. The results indicated HA gene may experience stronger positive selection compared to NA and matrix gene in process of adaptation to the human population globally. Out of 18 positive selected sites, the S220T (HA; found in Indian isolate) and I30V (NA; found in global isolate) were also reported in previous studies as clade VII specific markers [19]. Position A151T/V and R222K, are situated within A and D epitopic regions of HA and is also associated with receptor binding [22]. Since HA plays a crucial role in virus attachment, these substitution might have played an important role in virus transmission.

The present study is the first systematic study carried out to characterize the true genetic nature of recently circulating Indian H1N1pdm virus in post-pandemic phase. This study clearly indicates that the cosmopolitan clade VII is predominant in India. Few reported Clade VII markers revealed in this study indicates that the clade is undergone positive selection during virus evolution since last 3 years and a shift to clade VII in Indian isolates was observed from other circulating clades during 2009–2012. The complete genome information of recent H1N1pdm Indian virus isolate elucidated for the first time in this study will serve in future epidemiological surveillance in Indian subcontinent and abroad.

## Materials and Methods

### Clinical Samples and Virus

A total of 120 acute phase throat/nasopharyngeal swab samples suspected for H1N1pdm virus, with Influenza A like illness between 3–7 days of onset of fever (with case definition of sudden onset of fever >38°C, cough or sore throat) were referred from sentinel hospitals in Gwalior, India for the laboratory investigation of H1N1pdm outbreak during 2010 and 2011. Throat/nasopharyngeal swab samples were received in viral transport medium (Himedia) at appropriate cold temperature (4°C) and triple packaging system. All the samples were processed in the High Containment Facility (a biosafety level −3 laboratory) at DRDE, Gwalior. A total of four Indian isolates (3 from Gwalior and 1 from Bangalore) were selected for the complete genome sequence and phylogenetic analysis in this study. Out of positive samples, three viruses isolated from Gwalior, India (A/India/GWL-DSC/2010, A/India/GWL-01/2011, A/India/GWL-02/2011) and one virus isolated by Prof. V. Ravi, at NIMHANS, Bangalore (A/India/Blore/2010) were included for complete genome characterization.

### Nucleic Acid Extraction

Viral RNA was extracted from 140 µl of clinical sample and cell culture supernatant (Isolates) by using QIAamp viral RNA mini kit (Qiagen, Germany) in accordance with the manufacturer’s instructions. Finally, RNA was eluted in 50 µl of elution buffer and stored at −80°C until use.

### Real-time RT-PCR

The CDC Real-time RT-PCR assay was used for novel swine flu virus identification in MX 3000P quantitative PCR system (Stratagene, USA). The assay is based on Taqman chemistry including a panel of oligonucleotide primers and dual labeled hydrolysis probe sets [universal Influenza A (Inf A), swine influenza A (swInf A), swine H1 (swH1), and RNaseP (RP)] employing Invitrogen SuperScript^TM^III Platinum® one step quantitative kit. The amplification was carried out in a 25 µl reaction volume according to the CDC instruction and standard thermal profile for sample screening [23]. Briefly, the reagents include 2× buffer (Invitrogen One-step RT-PCR kit, USA) 12.5 µl, enzyme mix 0.5 µl, both forward and reverse primers 0.5 µl (40 µM), and probe 0.5 µl (10 µM) each and DEPC treated water added up to a total volume of 25 µl. Finally, 5 µl of viral RNA eluate extracted from different samples was added for Real-time RT-PCR assay.

### H1N1pdm Virus Isolation and Molecular Characterization

All the clinical samples were processed in Biosafety Level −3 Laboratory. Madin Darby Canine Kidney (MDCK) cells purchased from NCCS, Pune were maintained in Modified Eagle’s Medium (MEM) (Sigma-Aldrich, St. Louis, MO) supplemented with 5% fetal bovine serum (FBS) (Sigma-Aldrich) at 37°C in a humidified 5% CO_2_ atmosphere. The clinical samples (throat and nasal swabs) obtained from patients were inoculated in MDCK cell lines at 90% confluency for virus isolation as per standard protocol [24]. Tissue culture fluid was harvested after observing MDCK cell lines for cytopathic effect. Morphological changes of MDCK cells were photographed with an inverted microscope (Olympus IX 71) at 0 to 72 hr. The presence of pandemic H1N1 virus in infected culture fluid was demonstrated by hemagglutination, immunofluorescence using virus specific antibodies and CDC real time RT-PCR.

Hemagglutination (HA) test was performed using guinea pig RBC following standard protocol [25]. Briefly, the infected culture supernatant was allowed to react with 0.5% of RBC to hemagglutination reaction for 1 h at room temperature. After incubation, results were interpreted accordingly, a positive reaction was observed by mat formation in U-bottom plate (Nunc, USA) and settled RBCs in the form of button for negative reaction. For the immunofluorescence test (IFT), virus was allowed to infect the cells at required time points and the cells were washed 3 times with PBS followed by the fixation with chilled methanol for 1 h. The fixed cells were then permeabilized by 0.1% Triton-X 100 at room temperature for 20 min and incubated with rabbit Anti-pdmH1N1 HA pAb (1∶2000) (GenScript, USA) followed by anti-rabbit IgG-FITC conjugate (Sigma)(1∶160). Cells were washed and visualized under Carl-Zeiss Aximot 2 (Germany) microscope equipped for incident illumination with a narrow band filter combination selective for FITC. Virus at different passage levels were also confirmed by CDC Real time RT-PCR as described above.

### Complete Génome Amplification

One step RT-PCR was carried out to amplify all the eight segments using the recommended WHO-CDC whole genome primers [23]. Each gene segments were amplified in three to eight fragments of 324 to 833 bp (Minimum to maximum product size) with 100 bp overlapping sequence in order to get at least four fold sequence coverage. A total of 46 overlapping amplicons spanning the complete genomic region were amplified using 92 primers. To amplify each segment, 5 µl of RNA was added to a 25 µl of master mix containing 2.5 µl 10X PCR buffer, 1.5 µl MgCl_2_ (3 mM), 0.5 µl dNTP (200 µM each), 0.5 Reverse Transcriptase (0.4 units/µl), 0.5 µl RNAse inhibitor (0.4 units/µl), 0.5 µl TaqDNA polymerase (0.05 units/µl), 0.25 µl of respective forward and reverse primers and 13 µl of molecular biology grade water. The One-step RT-PCR was carried out using Enhanced Avian HS RT-PCR kit (Sigma, USA). The PCR amplification was carried out in a final volume of 25 µl in a thermal cycler (Bio-Rad, USA). The thermal profile comprised of reverse transcription at 48°C for 45 min, initial denaturation at 95°C for 2 min followed by 35 cycles at 95°C for 1 min, annealing at 56–65°C for 1 min, extension at 72°C for 2 min and final extension at 72°C for 10 min. The PCR products were gel purified from 1% agarose gel using the QIAquick gel extraction kit (Qiagen, Germany) and used as template in sequencing reactions.

### Sequencing Reaction

Double pass sequencing was carried out employing big dye terminator cycle sequencing ready reaction kit (Perkin-Elmer, Applied Biosystems, USA) on an ABI 310 sequencer. Briefly, each sequencing reaction was carried out in a final volume of 10 µl by mixing the Big Dye terminator mix containing the thermostable Ampli*Taq* DNA polymerase, dNTPs and four dye-labelled dideoxy nucleotide terminators (ddNTPs) and 25 ng of purified PCR product, and 3.2 pmol of either sense or antisense primer. Cycle sequencing parameters were as follows: 25 cycles of 96°C for 5 sec, 50°C for 15 sec, and 60°C for 4 min). The reaction mixture was column purified and the DNA was dried in vacuum. The DNA pellet was resuspended in 15 µl of hidiformamide, heated at 95°C for 5 min before loaded on the ABI 310 automated DNA sequencer (Applied Biosystems, USA).

### Sequence Analysis

The nucleotide sequences were retrieved, edited and analysed using the *SeqScape* (Applied Biosystems, USA) and *EditSeq* and *MegAlign* modules of Lasergene 5 software package (DNASTAR Inc, USA). Multiple sequence alignment was carried out employing MUSCLE [26]. The deduced amino acid was determined from the nucleotide sequence using the *EditSeq* module of Lasergene 5 software package (DNASTAR Inc, USA). The percent nucleotide identity and percent amino acid identity values were calculated as pairwise p-distances. Extensive phylogenetic analysis based on full HA gene (1701 nt) and complete genome (13158nt: concatenated eight segments) were carried out by including 45 and 65 globally diverse H1N1pdm sequences (Table S1) respectively using MrBayes version 3.1.2 [27]. The Bayesian tree was inferred by running a Markov-chain Monte Carlo algorithm for 1, million generations, sampling at every 100^th^ generation with a burn in setting of 10% of generations. The GTR+G+I model (general time-reversible model with gamma-distributed rates of variation among sites and a proportion of invariable sites) was found to be the best-fit model for our dataset. Convergence was assessed using mean SD in partition frequency values by using a threshold of 0.01.

### Selection Pressure Analysis

Selection pressure analysis acting on the codons of surface proteins i.e. hemagglutinin (HA), neuraminidase (NA) and matrix protein (MP) of H1N1pdm virus was carried out using HyPhy open-source software package available under the datamonkey web-server (http://www.datamonkey.org/) [28]. Analysis was performed using reference sequences [n = 80(HA); n = 73(NA); n = 71(MP)] including Indian H1N1pdm virus for all the three gene segments (Table S2). A separate analysis for HA and NA gene were also carried out by including 17 Indian H1N1pdm viruses (Table S2). The ratio of non-synonnymous (dN) to synonymous (dS) substitutions per site (dN/dS or ω) were estimated using five different approaches including: single likelihood ancestor counting (SLAC), fixed effects likelihood (FEL), random effects method (REL), mixed effects model of evolution (MEME), fast unbiased bayesian approximation (FUBAR). Best nucleotide substitutions model for different data sets as determined through the available tool in Datamonkey server was adopted in the analysis.

## Supporting Information

Table S1 **Gene bank accession numbers used in Phylogenetic analysis.**
(DOC)

Table S2 **Gene bank accession numbers used in selection pressure analysis of HA, NA and Matrix protein gene.**
(DOC)

Table S3 **Selection pressure analysis of HA protein (566 codons); NA protein (469 codons), Protein of Indian H1N1pdm virus using SLAC, FEL,REL,MEME and FUBAR methods. (** www.datamonkey.org **).**
(DOC)
