# Supplementary material for: Molecular Epidemiology and Complete Genome Characterization of H1N1pdm Virus from India
Source: PLoS One. 2013 Feb 15;8(2):e56364. doi: 10.1371/journal.pone.0056364 (PMC3574146; doi:10.1371/journal.pone.0056364)
Supplement: Table S1 — Gene bank accession numbers used in Phylogenetic analysis. (DOC) [file pone.0056364.s001.doc]

**Table S- 1:** GenBank accession numbers used in Phylogenetic analysis.

| S. no. Strain | | | | Accession number | | | |  | | | |
| --- | --- | --- | --- | --- | --- | --- | --- | --- | --- | --- | --- |
|  |  | PB2 | PB1 | | PA | HA | NP | | NA | MP | NS |
| 1. | A/California/04/2009 | FJ966079 | FJ966080 | | FJ966081 | **FJ966082** | FJ966083 | | FJ966084 | FJ966085 | FJ966086 |
| 2. | A/Pune/NIV6447/2009 | GU292361 | GU292367 | | GU292373 | **GU292353** | GU292379 | | GU292385 | GU292391 | GU292397 |
| 3 | A/India/Blore/2010 | JF265677 | JF764086 | | JF265676 | **JF293316** | JF265674 | | JF265672 | JF764082 | JF764084 |
| 4 | A/India/GWL_DSC/2010 | JF265678 | JF764085 | | JF265675 | **JF293315** | JF265673 | | JF265671 | JF510037 | JF764083 |
| 5 | A/India/GWL01/2011 | JX262203 | JX262205 | | JX262207 | **JQ319658** | JX262209 | | JX262201 | JX262211 | JX262213 |
| 6 | A/India/GWL02/2011 | JX262204 | JX262206 | | JX262208 | **JQ319657** | JX262210 | | JX262202 | JX262212 | JX262214 |
| 7 | A/California/07/2009 | FJ969530 | FJ969531 | | FJ969529 | **FJ969540** | FJ969536 | | GQ377078 | FJ969537 | FJ969528 |
| 8 | A/Canada-AB/RV1644/2009 | GQ465667 | GQ465751 | | GQ465739 | GQ465679 | GQ465715 | | GQ465702 | GQ465691 | GQ465727 |
| 9 | A/California/06/2009 | FJ966963 | FJ966965 | | FJ966964 | FJ966960 | FJ966961 | | FJ971075 | FJ966962 | FJ971074 |
| 10 | A/Blore/NIV236/2009 | GU292357 | GU292363 | | GU292369 | **GU292346** | GU292375 | | GU292381 | GU292387 | GU292393 |
| 11 | A/Blore/NIV310/2009 | GU292364 | GU292358 | | GU292370 | **GU292347** | GU292376 | | GU292382 | GU292388 | GU292394 |
| 12 | A/New York/3324/2009 | CY043202 | CY043201 | | CY043200 | **CY043195** | CY043198 | | CY043197 | CY043196 | CY043199 |
| 13 | A/Pune/NIV8489/2009 | GU292362 | GU292368 | | GU292374 | **GU292354** | GU292380 | | GU292386 | GU292392 | GU292398 |
| 14 | A/Shanghai/143T/2009 | GQ340061 | GQ340062 | | GQ411906 | **GQ411907** | GQ411909 | | GQ411905 | GQ340064 | GQ340063 |
| 15 | A/Pune/NIV6196/2009 | GU292360 | GU292366 | | GU292372 | **GU292352** | GU292378 | | GU292384 | GU292390 | GU292396 |
| 16 | A/Wisconsin/629-D00008/2009 | CY051054 | CY051053 | | CY051052 | **CY051047** | CY051050 | | CY051049 | CY051048 | CY051051 |
| 17 | A/Beijing/3/2009 | GQ225378 | GQ225379 | | GQ225380 | **GQ225381** | GQ225382 | | GQ225383 | GQ225384 | GQ225385 |
| 18 | A/Hyd/NIV51/2009 | GU292359 | GU292365 | | GU292371 | **GU292350** | GU292377 | | GU292383 | GU292389 | GU292395 |
| 19 | A/Osaka/1/2009 | GQ222055 | GQ222046 | | GQ222037 | **GQ219578** | GQ223421 | | GQ220734 | GQ222028 | GQ223430 |
| 20 | A/Korea/01/2009 | GQ160811 | GQ160813 | | GQ160812 | **GQ131023** | GQ131024 | | GQ132185 | GQ131025 | GQ131026 |
| 21 | A/England/195/2009 | GQ166656 | GQ166655 | | GQ166654 | **GQ166661** | GQ166658 | | GQ166659 | GQ166660 | GQ166657 |
| 22 | A/Hamburg/4/2009 |  |  | |  | **GQ166213** |  | |  |  |  |
| 23 | A/New York/3177/2009 | CY041604 | CY041603 | | CY041602 | **CY041597** | CY041600 | | CY041599 | CY041598 | CY041601 |
| 24 | A/Kansas/03/2009 |  |  | |  | **GQ168644** |  | |  |  |  |
| 25 | A/Moscow/WRAIR4316N/2011 | CY098049 | CY098050 | | CY098051 | CY098052 | CY098053 | | CY098054 | CY098055 | CY098056 |
| 26 | A/Netherlands/602/2009 | CY046940 | CY046941 | | CY046942 | CY039527 | CY046943 | | CY039528 | CY046944 | CY046945 |
| 27 | A/Santo Domingo/0573N/2009 | CY041980 | CY041981 | | CY041982 | CY041983 | CY041984 | | CY041985 | CY041986 | CY041987 |
| 28 | A/Brawley/40081/2009 | CY043083 | CY043084 | | CY043085 | CY043086 | CY043087 | | CY043088 | CY043089 | CY043090 |
| 29 | A/Vladivostok/01/2009 | GU211226 | GU211225 | | GU211224 | GU211219 | GU211222 | | GU211221 | GU211220 | GU211223 |
| 30 | A/Craven/WR0019/2009 | CY049817 | CY049818 | | CY049819 | CY049820 | CY049821 | | CY049822 | CY049823 | CY049824 |
| 31 | A/Nanjing/2/2009 | GQ455029 | GQ455030 | | GQ455031 | GQ455032 | GQ455033 | | GQ455034 | GQ455035 | GQ455036 |
| 32 | A/Nebraska/02/2009 | GQ200260 | GQ168875 | | GQ457496 | GQ377082 | GQ117104 | | GQ221802 | GQ457495 | GQ117106 |
| 33 | A/Wisconsin/629-D00022/2009 | CY051230 | CY051229 | | CY051228 | CY051223 | CY051226 | | CY051225 | CY051224 | CY051227 |
| 34 | A/Colorado/03/2009 | GQ200263 | GQ168884 | | GQ200264 | GQ117119 | GQ117117 | | GQ221813 | GQ457502 | GQ377090 |
| 35 | A/Sichuan/1/2009 | GQ166228 | GQ166227 | | GQ166226 | GQ166223 | GQ166225 | | GQ166224 | GQ166229 | GQ166230 |
| 36 | A/Minnesota/02/2009 | GQ117070 | GQ117069 | | GQ457488 | GQ338364 | GQ117068 | | GQ117071 | GQ117073 | GQ117072 |
| 37 | A/Indiana/09/2009 | GQ168870 | GQ117093 | | GQ117095 | GQ117097 | GQ117092 | | GQ117094 | GQ117096 | GQ168871 |
| 38 | A/Amagasaki/1/2009 | GQ222050 | GQ222041 | | GQ222032 | GQ219574 | GQ223416 | | GQ220730 | GQ222023 | GQ223425 |
| 39 | A/Sakai/1/2009 | GQ267845 | GQ267844 | | GQ267843 | GQ267839 | GQ267841 | | GQ261274 | GQ267840 | GQ267842 |
| 40 | A/Himeji/1/2009 | GQ267838 | GQ267837 | | GQ267836 | GQ261272 | GQ267834 | | GQ261273 | GQ267833 | GQ267835 |
| 41 | A/Kobe/1/2009 | GQ222054 | GQ222045 | | GQ222036 | GQ219577 | GQ223420 | | GQ220733 | GQ222027 | GQ223429 |
| 42 | A/Beijing/501/2009 | GQ223412 | GQ223413 | | GQ223414 | GQ223408 | GQ223410 | | GQ223415 | GQ223409 | GQ223411 |
| 43 | A/New York/4735/2009 | CY051670 | CY051669 | | CY051668 | CY051663 | CY051666 | | CY051665 | CY051664 | CY051667 |
| 44 | A/Canada-PQ/RV1758/2009 | GQ465668 | GQ465752 | | GQ465740 | GQ465680 | GQ465716 | | GQ465703 | GQ465692 | GQ465728 |
| 45 | A/Utsunomiya/1/2009 | GQ334354 | GQ334361 | | GQ334360 | GQ334355 | GQ334358 | | GQ334357 | GQ334356 | GQ334359 |
| 46 | A/Hunan/SWL3/2009 | GQ463197 | GQ463198 | | GQ463199 | GQ463200 | GQ463201 | | GQ463202 | GQ463203 | GQ463204 |
| 47 | A/Thailand/CU-B5/2009 | GQ866948 | GQ866949 | | GQ866950 | GQ866951 | GQ866952 | | GQ866953 | GQ866954 | GQ866955 |
| 48 | A/Taiwan/T1773/2009 | CY044217 | CY044218 | | CY044219 | CY044220 | CY044221 | | CY044222 | CY044223 | CY044224 |
| 49 | A/Silver Spring/SP509/2009 | CY044176 | CY044177 | | CY044178 | CY044179 | CY044180 | | CY044181 | CY044182 | CY044183 |
| 50 | A/Nanjing/3/2009 | GU198198 | GU198199 | | GU198200 | GU198201 | GU198202 | | GU198203 | GU198204 | GU198205 |
| 51 | A/Shizuoka/759/2009 | GQ334352 | GQ334353 | | GQ334351 | GQ334346 | GQ334349 | | GQ334348 | GQ334347 | GQ334350 |
| 52 | A/Shiga/3/2009 | GQ324571 | GQ324570 | | GQ324569 | GQ287623 | GQ324567 | | GQ287624 | GQ324566 | GQ324568 |
| 53 | A/Mexico City/WR1100N/2009 | CY049992 | CY049993 | | CY049994 | CY049995 | CY049996 | | CY049997 | CY049998 | CY049999 |
| 54 | A/San Salvador/0169T/2009 | CY049888 | CY049889 | | CY049890 | CY049891 | CY049892 | | CY049893 | CY049894 | CY049895 |
| 55 | A/New Bern/WR0670/2009 | CY049920 | CY049921 | | CY049922 | CY049923 | CY049924 | | CY049925 | CY049926 | CY049927 |
| 56 | A/Cherry Point/WR0100/2009 | CY049856 | CY049857 | | CY049858 | CY049859 | CY049860 | | CY049861 | CY049862 | CY049863 |
| 57 | A/Taiwan/1018/2011 | JN187172 | JN187230 | | JN187259 | JN187143 | JN187288 | | JN187201 | JN187317 | JN187346 |
| 58 | A/Boston/DOA14/2011 | CY111213 | CY111212 | | CY111211 | CY111206 | CY111209 | | CY111208 | CY111207 | CY111210 |
| 59 | A/Sydney/DD3-58/2011 | CY092863 | CY092862 | | CY092861 | CY092856 | CY092859 | | CY092858 | CY092857 | CY092860 |
| 60 | A/Thailand/CU-H2911/2011 | CY089460 | CY089461 | | CY089462 | CY089463 | CY089464 | | CY089465 | CY089466 | CY089467 |
| 61 | A/Mexico/InDRE3740/2011 | CY120019 | CY120020 | | CY116630 | CY116642 | CY115447 | | CY115448 | CY115449 | CY115450 |
| 62 | A/Missouri/NHRC0001/2011 | CY092424 | CY092423 | | CY092422 | CY092417 | CY092420 | | CY092419 | CY092418 | CY092421 |
| 63 | A/Cheboksary/IIV-92/2011 | JN703379 | JN703380 | | JN704790 | JN704791 | JN704792 | | JN704793 | JN704794 | JN704795 |
| 64 | A/Tomsk/IIV-19/2012 | JQ768350 | JQ768351 | | JQ768352 | JQ768353 | JQ768354 | | JQ768355 | JQ768356 | JQ768357 |
| 65 | A/California/NHRC0001/2011 | CY092887 | CY092886 | | CY092885 | CY092880 | CY092883 | | CY092882 | CY092881 | CY092884 |
| 66 | A/Brazil/AVS08/2011 | CY120754 | CY120753 | | CY120752 | CY120747 | CY120750 | | CY120749 | CY120748 | CY120751 |
| 67 | A/South Carolina/09/2009 | GQ200222 | GQ168854 | | GQ457472 | GQ117056 | GQ117052 | | GQ221795 | GQ221796 | GQ117054 |
| 68 | A/District of Columbia/WRAIR313/2011 |  |  | |  | **CY090027** |  | |  |  |  |
| 69 | A/Nizhnii Novgorod/CRIE- BLM/2011 |  |  | |  | **JN714492** |  | |  |  |  |
| 70 | A/Rio Grande do Sul/361/2011 |  |  | |  | **CY100002** |  | |  |  |  |
| 71 | A/St.Petersburg/CRIE- GoVM/2011 |  |  | |  | **JN714517** |  | |  |  |  |
| 72 | A/Shanghai/3162T/2011 |  |  | |  | **JN631050** |  | |  |  |  |
| 73 | A/Bangkok/INS520/2010 |  |  | |  | **CY098563** |  | |  |  |  |
| 74 | A/Volgograd/CRIE-DMV/2011 |  |  | |  | **JN714508** |  | |  |  |  |
| 75 | A/Finland/65/2011 |  |  | |  | **JN601109** |  | |  |  |  |
| 76 | A/Assam/2220/2009 |  |  | |  | **JN600356** |  | |  |  |  |
| 77 | A/Assam/2590/2010 |  |  | |  | **JN600357** |  | |  |  |  |
| 78 | A/Cambodia/U127/2010 |  |  | |  | **JN588791** |  | |  |  |  |
| 79 | A/Netherlands/2631_1202/2010 |  |  | |  | **JF906183** |  | |  |  |  |
| 80 | A/Delhi/NIV3704/2009 |  |  | |  | **GU292349** |  | |  |  |  |
| 81 | A/Pune/NIV9355/2009 |  |  | |  | **GU292355** |  | |  |  |  |
| 82 | A/Pune/NIV10278/2009 |  |  | |  | **GU292344** |  | |  |  |  |
| 83 | A/Mum/NIV9945/2009 |  |  | |  | **GU292356** |  | |  |  |  |
| 84 | A/Omsk/02/2009 |  |  | |  | **GU211235** |  | |  |  |  |
| 85 | A/Delhi/NIV3610/2009 |  |  | |  | **GU292348** |  | |  |  |  |
| 86 | A/Denmark/523/2009 |  |  | |  | **CY043334** |  | |  |  |  |
| 87 | A/Ohio/07/2009 |  |  | |  | **GQ117100** |  | |  |  |  |
| 88 | A/Mum/NIV5442/2009 |  |  | |  | **GU292351** |  | |  |  |  |
| 89 | A/Shanghai/1/2009 |  |  | |  | **GQ225357** |  | |  |  |  |
| 90 | A/Pune/NIV10604/2009 |  |  | |  | **GU292345** |  | |  |  |  |

Note: GenBank accession number used in HA gene based phylogeny are highlighted with bold font.
