# Supplementary material for: Molecular Epidemiology and Complete Genome Characterization of H1N1pdm Virus from India
Source: PLoS One. 2013 Feb 15;8(2):e56364. doi: 10.1371/journal.pone.0056364 (PMC3574146; doi:10.1371/journal.pone.0056364)
Supplement: Table S2 — Gene bank accession numbers used in selection pressure analysis of HA, NA and Matrix protein gene. (DOC) [file pone.0056364.s002.doc]

**Table S- 2:** GenBank accession numbers used in selection pressure analysis of HA, NA and Matrix protein gene.

| S.No. | Strain | Accession Number  HA NA MP | | |
| --- | --- | --- | --- | --- |
| 1 | A/California/04/2009 | FJ966082 | FJ966084 | FJ966085 |
| 2 | A/California/07/2009 | FJ969540 | GQ377078 | FJ969537 |
| 3 | A/Canada-AB/RV1644/2009 | GQ465679 | GQ465702 | GQ465691 |
| 4 | A/California/06/2009 | FJ966960 | FJ971075 | FJ966962 |
| 5 | A/California/07/2009 | FJ969540 | GQ377078 | FJ969537 |
| 6 | A/Canada-AB/RV1644/2009 | GQ465679 | GQ465702 | GQ465691 |
| 7 | A/California/06/2009 | FJ966960 | FJ971075 | FJ966962 |
| 8 | A/Omsk/02/2009 | GU211235 | GU211237 | GU211236 |
| 9 | A/Denmark/523/2009 | CY043334 | CY043336 | CY043337 |
| 10 | A/Shanghai/1/2009 | GQ225357 | GQ225359 | GQ225360 |
| 11 | A/New York/3324/2009 | CY043195 | CY043197 | CY043196 |
| 12 | A/Shanghai/143T/2009 | GQ411907 | GQ411905 | GQ340064 |
| 13 | A/Wisconsin/629-D00008/2009 | CY051047 | CY051049 | CY051048 |
| 14 | A/Beijing/3/2009 | GQ225381 | GQ225383 | GQ225384 |
| 15 | A/Osaka/1/2009 | GQ219578 | GQ220734 | GQ222028 |
| 16 | A/Korea/01/2009 | GQ131023 | GQ132185 | GQ131025 |
| 17 | A/England/195/2009 | GQ166661 | GQ166659 | GQ166660 |
| 18 | A/Hamburg/4/2009 | GQ166213 | GQ166217 | GQ166219 |
| 19 | A/New York/3177/2009 | CY041597 | CY041599 | CY041598 |
| 20 | A/Kansas/03/2009 | GQ168644 | GQ168643 | GQ168858 |
| 21 | A/Moscow/WRAIR4316N/2011 | CY098052 | CY098054 | CY098055 |
| 22 | A/Netherlands/602/2009 | CY039527 | CY039528 | CY046944 |
| 23 | A/Santo Domingo/0573N/2009 | CY041983 | CY041985 | CY041986 |
| 24 | A/Brawley/40081/2009 | CY043086 | CY043088 | CY043089 |
| 25 | A/Vladivostok/01/2009 | GU211219 | GU211221 | GU211220 |
| 26 | A/Craven/WR0019/2009 | CY049820 | CY049822 | CY049823 |
| 27 | A/Nanjing/2/2009 | GQ455032 | GQ455034 | GQ455035 |
| 28 | A/Nebraska/02/2009 | GQ377082 | GQ221802 | GQ457495 |
| 29 | A/Wisconsin/629-D00022/2009 | CY051223 | CY051225 | CY051224 |
| 30 | A/Colorado/03/2009 | GQ117119 | GQ221813 | GQ457502 |
| 31 | A/Sichuan/1/2009 | GQ166223 | GQ166224 | GQ166229 |
| 32 | A/Minnesota/02/2009 | GQ338364 | GQ117071 | GQ117073 |
| 33 | A/Indiana/09/2009 | GQ117097 | GQ117094 | GQ117096 |
| 34 | A/Amagasaki/1/2009 | GQ219574 | GQ220730 | GQ222023 |
| 35 | A/Sakai/1/2009 | GQ267839 | GQ261274 | GQ267840 |
| 36 | A/Himeji/1/2009 | GQ261272 | GQ261273 | GQ267833 |
| 37 | A/Kobe/1/2009 | GQ219577 | GQ220733 | GQ222027 |
| 38 | A/Beijing/501/2009 | GQ223408 | GQ223415 | GQ223409 |
| 39 | A/Utsunomiya/1/2009 | GQ334355 | GQ334357 | GQ334356 |
| 40 | A/Hunan/SWL3/2009 | GQ463200 | GQ463202 | GQ463203 |
| 41 | A/Netherlands/2631_1202/2010 | JF906183 |  |  |
| 42 | A/Ohio/07/2009 | GQ117100 | GQ323479 |  |
| 43 | A/Shanghai/3162T/2011 | JN631050 | JN631044 |  |
| 44 | A/Volgograd/CRIE-DMV/2011 | JN714508 |  |  |
| 45 | A/Finland/65/2011 | JN601109 |  |  |
| 46 | A/Assam/2220/2009 | JN600356 |  |  |
| 47 | A/Assam/2590/2010 | JN600357 |  |  |
| 48 | A/Cambodia/U127/2010 | JN588791 |  |  |
| 49 | A/Thailand/CU-B5/2009 | GQ866951 | GQ866953 | GQ866954 |
| 50 | A/Taiwan/T1773/2009 | CY044220 | CY044222 | CY044223 |
| 51 | A/Silver Spring/SP509/2009 | CY044179 | CY044181 | CY044182 |
| 52 | A/Nanjing/3/2009 | GU198201 | GU198203 | GU198204 |
| 53 | A/Shizuoka/759/2009 | GQ334346 | GQ334348 | GQ334347 |
| 54 | A/Shiga/3/2009 | GQ287623 | GQ287624 | GQ324566 |
| 55 | A/San Salvador/0169T/2009 | CY049891 | CY049893 | CY049894 |
| 56 | A/Cherry Point/WR0100/2009 | CY049859 | CY049861 | CY049862 |
| 57 | A/Taiwan/1018/2011 | JN187143 | JN187201 | JN187317 |
| 58 | A/Boston/DOA14/2011 | CY111206 | CY111208 | CY111207 |
| 59 | A/Thailand/CU-H2911/2011 | CY089463 | CY089465 | CY089466 |
| 60 | A/Mexico/InDRE3740/2011 | CY116642 | CY115448 | CY115449 |
| 61 | A/California/NHRC0001/2011 | CY092880 | CY092882 | CY092881 |
| 62 | A/Brazil/AVS08/2011 | CY120747 | CY120749 | CY120748 |
| 63 | A/South Carolina/09/2009 | GQ117056 | GQ221795 | GQ221796 |
| 64 | **A/Pune/NIV6447/2009** | **GU292353** | **GU292385** | **GU292391** |
| 65 | **A/India/Blore/2010** | **JF293316** | **JF265672** | **JF764082** |
| 66 | **A/India/GWL_DSC/2010** | **JF293315** | **JF265671** | **JF510037** |
| 67 | **A/India/GWL01/2011** | **JQ319658** | **JX262201** | **JX262211** |
| 68 | **A/India/GWL02/2011** | **JQ319657** | **JX262202** | **JX262212** |
| 69 | **A/Delhi/NIV3704/2009** | **GU292349** |  |  |
| 70 | **A/Pune/NIV9355/2009** | **GU292355** | **CY088703** | **CY088704** |
| 71 | **A/Blore/NIV236/2009** | **GU292346** | **GU292381** | **GU292387** |
| 72 | **A/Pune/NIV10278/2009** | **GU292344** | **CY088640** | **CY088641** |
| 73 | **A/Mum/NIV9945/2009** | **GU292356** | **CY088710** | **CY088711** |
| 74 | **A/Delhi/NIV3610/2009** | **GU292348** | **CY088675** | **CY088676** |
| 75 | **A/Mum/NIV5442/2009** | **GU292351** | **CY088689** | **CY088690** |
| 76 | **A/Blore/NIV310/2009** | **GU292347** | **GU292382** | **GU292388** |
| 77 | **A/Pune/NIV8489/2009** | **GU292354** | **GU292386** | **GU292392** |
| 78 | **A/Pune/NIV6196/2009** | **GU292352** | **GU292384** | **GU292390** |
| 79 | **A/Pune/NIV10604/2009** | **GU292345** | **HM241726** | **HM241727** |
| 80 | **A/Hyd/NIV51/2009** | **GU292350** | **GU292383** | **GU292389** |

Note: GenBank accession number of Indian Isolates used in selection pressure analysis were highlighted in bold font.
