# Supplementary material for: Molecular Epidemiology and Complete Genome Characterization of H1N1pdm Virus from India
Source: PLoS One. 2013 Feb 15;8(2):e56364. doi: 10.1371/journal.pone.0056364 (PMC3574146; doi:10.1371/journal.pone.0056364)
Supplement: Table S3 — Selection pressure analysis of HA protein (566 codons); NA protein (469 codons), Protein of Indian H1N1pdm virus using SLAC, FEL,REL,MEME and FUBAR methods. ( www.datamonkey.org ). (DOC) [file pone.0056364.s003.doc]

**Table S-3:** Selection pressure analysis of HA and NA Protein of Indian H1N1pdm virus.

| **Protein** | **Codon** | **SLAC** | | **FEL** | | **REL** | | **MEME** | | **FUBAR** | |
| --- | --- | --- | --- | --- | --- | --- | --- | --- | --- | --- | --- |
|  |  | **dN-dS** | **p-value** | **dN-dS** | **p-value** | **dN-dS** | **Bayes Factor** | **ω^+^** | **p-value** | **dN-dS** | **Post. Pr.** |
| **HA** | 220 | 8.76 | 0.43 | 77.46 | 0.202 | 0.560 | 25.135 | >100 | 0.224 | 1.301 | 0.838 |
|  | 278 | 8.362 | 0.444 | 64.515 | 0.212 | 0.553 | 23.012 | >100 | 0.233 | 1.047 | 0.825 |
|  | 391 | 11.669 | 0.451 | 107.40 | 0.143 | 0.728 | 120.47 | >100 | 0.074 | 3.641 | 0.950 |
| **NA** | 81 | 11.87 | 0.203 | 26.64 | 0.091 | -0.039 | 1.000 | >100 | 0.124 | 2.914 | 0.952 |
|  | 248 | 10.659 | 0.313 | 33.551 | 0.124 | -0.039 | 1.000 | >100 | 0.073 | 4.415 | 0.961 |

Note: The sites found under positive selection by atleast two methods are shown*.

*** Significance value**

SLAC P value—0.5

FEL P value- 0.3

REL Bayes factor- 50

MEME P value- 0.1

FUBAR Posterior probability- 0.9
